# Functional Nanomaterials for Sensing and Detection

**DOI:** 10.3390/nano14010128

**Published:** 2024-01-04

**Authors:** Weiping Cai

**Affiliations:** Key Laboratory of Materials Physics, Anhui Key Laboratory of Nanomaterials and Nanotechnology, Institute of Solid State Physics, HFIPS, Chinese Academy of Sciences, Hefei 230031, China; wpcai@issp.ac.cn

Functional nanomaterials involve various nanostructured objects, such as zero-dimensional (0D), 1D, and 2D nano-objects (nanoparticles, nanowires, nanotubes, nanosheets, etc.), as well as materials with nanostructured surfaces, including metals, semiconductors, and organic materials. These nanomaterials possess a high surface/volume ratio, as well as nanotip- and nanogap-induced physical effects, which lead to functional properties that significantly differ from the nanomaterials’ bulk properties and hence afford them with great potential applications in sensing and detection. These functional nanomaterials for sensing and detection are mostly used as transducers in devices such as spectral devices, chemiresistive sensors, and photodetection devices. They are also employed, in the design of nanosensors, as capture agents (e.g., magnetic nanoparticles) and signal amplifiers (plasmonic metals with nanopatterned surfaces for surface-enhanced Raman scattering (SERS) chips), as well as identification elements (polymers for molecular imprinting), in addition to other applications. This Special Issue covers the latest advances in the field of these functional nanomaterials for sensing and detection. 

Via a sacrificial template approach, H. Zhang et al. fabricated a two-dimensional honeycomb-like ordered porous ZnO/WO_3_ composite sensing matrix on a commercial ceramic tube with a curved and rough surface as a high-performance chemiresistive sensor, and they achieved efficient detection of trace volatile organic compound gases with a considerably low detection limit [1], whereas Jolly Bhadra et al. designed two-dimensional (MoS_2_, WS_2_)/PANI nanocomposites and developed ammonia sensors that operate at room temperature and have high sensitivity [2]. Both presented new functional nanomaterials for gas sensing devices. Based on a liquid crystalline Zn(II) coordination complex, F. Manea et al. designed and fabricated nanostructured hybrid electrodes for use in a high-performance electrochemical hybrid sensor of uric acid, and they revealed great potential for the development of advanced electrochemical detection of uric acid [3]. 

Surface modification is an important route to achieving nanomaterial functionalization, especially for spectral sensors and sensing chips. E. Perret et al. developed a method for detecting the presence of functional molecules modified on silicon nanowires via the backscattered signal of a resonant structure, and they achieved the identification of a functionalization type of nanowires [4]. Q. Zhao et al. described the synthesis of hierarchical Au/CuS hybrids with high-density nanotips for ultra-sensitive SERS chips with good reproducibility and fast recyclability [5]. Localized surface plasmon resonance (LSPR)-based sensors are a very promising type of optical biosensor for label-free detection. In another contribution, J. Borges et al. developed a plasmonic Au-TiO_2_ thin film and demonstrated its potential use as an LSPR platform for label-free biosensors [6]. Fluorescence-based detection is one of main sensing techniques, and S. J Park et al. fabricated a nitrogen and phosphorus co-doped carbon dot/silver nanoparticle nanocomposite for a fluorescence sensor, which could sensitively detect metformin hydrochloride with high selectivity [7]. Efficient pH detection is important in chemical syntheses. G. Bokias et al. reported a water-soluble Ir(III)-contained copolymer and demonstrated characteristic pH-responsive color changes under ultraviolet illumination in the acidic pH region [8].

In this Special Issue, we also discuss the functional nanomaterials for ultraviolet and infrared photodetection. W. Sun et al. synthesized an oriented hexagonal boron nitride on gallium nitride via chemical vapor deposition for a vertical-ordered h-BN/GaN heterojunction photodetector, which exhibited high ultraviolet photoresponsivity [9]. In addition, T. He et al. summarized 2D/3D hybrid van der Waals heterojunctions for an infrared photodetector, and they discussed the prospects for the development of 2D/3D high-performance infrared detection technology [10].

In summary, this Special Issue highlights some recent studies discussing the latest progress in the application of functional nanomaterials for sensing and detection. I believe that these articles will be helpful to readers in this field of research. 

## References

[1] Lei B., Zhang H., Zhao Q., Liu W., Wei Y., Lu Y., Xiao T., Kong J., Cai W. (2023). Facile Synthesis of ZnO/WO_3_ Nanocomposite Porous Films for High-Performance Gas Sensing of Multiple VOCs. Nanomaterials.

[2] Parangusan H., Bhadra J., Al-Qudah R.A., Elhadrami E.C., Al-Thani N.J. (2022). Comparative Study on Gas-Sensing Properties of 2D (MoS_2_, WS_2_)/PANI Nanocomposites-Based Sensor. Nanomaterials.

[3] Negrea S., Andelescu A.A., Sorina Ilies S., Cretu C., Cseh L., Rastei M., Donnio B., Szerb E.I., Manea F. (2022). Design of Nanostructured Hybrid Electrodes Based on a Liquid Crystalline Zn(II) Coordination Complex-Carbon Nanotubes Composition for the Specific Electrochemical Sensing of Uric Acid. Nanomaterials.

[4] Requena F., Samuel Ahoulou S., Barbot N., Kaddour D., Nedelec J.M., Baron T., Perret E. (2022). Towards Wireless Detection of Surface Modification of Silicon Nanowires by an RF Approach. Nanomaterials.

[5] Fu H., Liu W., Li J., Wu W., Zhao Q., Bao H., Zhou L., Zhu S., Kong J., Zhang H. (2022). High-Density-Nanotips-Composed 3D Hierarchical Au/CuS Hybrids for Sensitive, Signal-Reproducible, and Substrate-Recyclable SERS Detection. Nanomaterials.

[6] Pereira-Silva P., Meira D.D., Costa-Barbosa A., Costa D., Rodrigues M.S., Borges J., Machado A.V., Cavaleiro A., Sampaio P., Vaz F. (2022). Immobilization of Streptavidin on a Plasmonic Au-TiO_2_ Thin Film towards an LSPR Biosensing Platform. Nanomaterials.

[7] Le T.H., Kim J.H., Park S.J. (2022). A Co-Doped Carbon Dot/Silver Nanoparticle Nanocomposite-Based Fluorescence Sensor for Metformin Hydrochloride Detection. Nanomaterials.

[8] Tsakaraki D., Andreopoulou A.K., Bokias G. (2022). pH-Responsive Emission of Novel Water-Soluble Polymeric Iridium(III) Complexes. Nanomaterials.

[9] Peng Y., Yang Y.F., Xiao K., Yang Y.L., Ding H., Deng J., Sun W. (2023). Direct Synthesis of Vertical Self-Assembly Oriented Hexagonal Boron Nitride on Gallium Nitride and Ultrahigh Photoresponse Ultraviolet Photodetectors. Nanomaterials.

[10] Tang Q., Zhong F., Li Q., Weng J., Li J., Lu H., Wu H., Liu S., Wang J., Deng K. (2023). Infrared Photodetection from 2D/3D van der Waals Heterostructures. Nanomaterials.

